# Editorial: Advances in organ-specific autoimmune response: from basics to clinics

**DOI:** 10.3389/fimmu.2024.1394736

**Published:** 2024-03-15

**Authors:** Fan Xiao, Xuming Tang, Kongyang Ma, Xiaoyan Dai

**Affiliations:** ^1^ Department of Pathology, Li Ka Shing Faculty of Medicine, The University of Hong Kong, Hong Kong, Hong Kong SAR, China; ^2^ National Heart, Lung, and Blood Institute, National Institutes of Health, Bethesda, MD, United States; ^3^ Centre for Infection and Immunity Studies, School of Medicine, The Sun Yat-sen University, Guangdong, Shenzhen, China; ^4^ Clinical Research Institute, the Second Affiliated Hospital, Hengyang Medical School, University of South China, Hengyang, China

**Keywords:** autoimmune disease, organ-specific responses, pathogenesis, diagnosis, therapy

Organ-specific autoimmune response is the key pathogenic event during the development of autoimmune diseases. Various factors including viral infections, genetic susceptibilities and dysregulated immune responses contribute to organ-specific immunity and promote tissue damage. It has been shown that many immune subsets including T and B cells infiltrate into target organs of patients with organ-specific autoimmune diseases, highlighting the important roles of tissue-specific autoimmune responses in disease pathogenesis (1, 2). Recent studies have revealed the extensive crosstalk between tissue cells and infiltrating immune cells, which drive organ-specific inflammation (3). Currently, effective therapies are still lacking for patients with autoimmune diseases, which is largely due to the limited understanding of tissue-specific autoimmune responses. Recent studies have shown that targeting disturbed cytokine network in organs might be an effective therapeutic strategy (4–6). Therefore, the studies of organ-specific immunity are important for understanding autoimmune disease pathogenesis and will facilitate the development of novel therapies. The current Research Topic focuses on deciphering the organ-specific responses in various autoimmune diseases and provides new insights into the cellular and molecular mechanisms and the clinical implications. As editors of the Research Topic, we present the major contributions of the accepted articles and discuss future perspectives in this important research field.

Primary Sjögren’s syndrome (pSS) is a chronic autoimmune disease characterized by dysfunction and inflammation of exocrine glands, including salivary glands and lacrimal glands. PSS patients usually show systemic complications with the involvement of multiple extraglandular organs, such as lungs, kidney, and joints (7). Various B and T cell subsets are detected in the inflamed organs of pSS patients. However, it is still unclear how these extraglandular organs are involved and the mechanisms of organ-specific immune responses during the development of pSS. Sato-Fukuba et al. investigated pulmonary lesions and compared glandular and extraglandular inflammation in a mouse model of pSS. Severe inflammatory lesions with lymphocytic infiltration were observed in both salivary glands and lung tissues of the NFS/sld mice that have undergone neonatal thymectomy, which represents a novel model of pSS. T cells were the major infiltrating population in salivary glands whereas both immunohistochemical analysis and immunofluorescence staining revealed dominant infiltration of B cells in the pulmonary lesions of the pSS mouse model. Flow cytometric analysis further showed increased numbers of B cells but comparable numbers of CD4 T cells in the lungs of control and pSS model mice, suggesting an important role of B cells in pulmonary inflammation. Unlike SG-infiltrating B cells, the lung-infiltrating B cells are CD23^+^ while their generation is dependent on the Th2 condition in the lungs. These novel findings revealed distinct B cell phenotypes in different organs involved in pSS development, highlighting the importance of organ-specific B and T cell responses in the pathogenesis of autoimmune diseases.

Histopathological and serological examinations are vital for the diagnosis and treatment of organ-specific autoimmune diseases. Hakroush et al. validated a recently described scoring system for short-term treatment response to therapeutic plasma exchange (PLEX) in a cohort of 53 patients with antineutrophil cytoplasmic antibody-associated vasculitis, a small vessel vasculitis affecting multiple organs. The enrolled patients also presented with rapidly progressive glomerulonephritis that was confirmed with histological analysis of kidney biopsies. This study suggests that PLEX scoring together with renal biopsy analysis may represent important prognostic value for predicting poor outcomes in the patient population, which warrants further investigation and validation in large cohorts. In organ-specific autoimmune diabetes mellitus (DM), the combined presence of autoantibodies against glutamic acid decarboxylase (GADA) and the islet-specific cation efflux transporter ZnT8 (ZnT8A) serves as an important parameter for predicting clinical manifestations. Compared with radioligand binding assays, current ELISA methods are less sensitive and specific. Trabucchi et al. developed a bridge ELISA method for simultaneous detection of GADA and ZnT8A in serum samples. They expressed and purified fusion protein of GADA/ZnT8A with immunoreactive conformation of the epitopes. The novel bridge ELISA immunoassay showed high sensitivity and specificity for the detection of ZnT8A and/or GADA and exhibited high accuracy in distinguishing between serum samples from healthy individuals and DM patients. Collectively, these newly developed clinical laboratory methods will be of important diagnostic value for patients with autoimmune diseases.

Due to the complexity of organ-specific immune responses during the development of autoimmune diseases, combinational therapies are gaining more and more attention with improved efficacy for treating many diseases. Granulomatous polyangiitis (GPA) is a rare autoimmune disease characterized by necrotizing granulomatous polyangiitis with the involvement of multiple organs. Huang et al. reported an interesting case of combination treatment with telitacicept, cyclophosphamide and glucocorticoids for a severe GPA patient involving multiple systems including kidneys, lungs, nose and ears. The patient showed much improved renal function as well as hearing and lung lesions upon the combinational therapy. Since telitacicept effectively suppresses the development and maturation of abnormal B cells by targeting BAFF and APRIL, the case report suggests an important role of B cell dysregulation in GPA patients with organ-threatening complications. More clinical trials will be needed to determine the safety and efficacy of telitacicept combined with cyclophosphamide and glucocorticoids for treating GPA patients.

The molecular mechanisms of autoimmune responses are largely unclear. Recently, emerging evidence has revealed the involvement of various factors including genetic variability, cytokines and miRNAs in organ-specific molecular events in different autoimmune diseases. In this Research Topic, two elegant review papers comprehensively discuss the current understanding of the engagement of distinct molecules, miRNAs, and immune cells in autoimmune disorders. Salfi et al. critically reviewed the available literature and identified important molecular pathways that contributed to the development of focal segmental glomerulosclerosis (FSGS), a histological pattern of kidney injury that affects glomerular tuft. Although the pathogenesis is unknown, available evidence suggests that immune dysregulation promotes kidney-specific injuries in FSGS. Salfi et al. summarized and discussed the important roles of immune cell populations and cytokines, such as IL-13, BAFF, CLCF1 *etc.* in the development of FSGS. Moreover, Salfi et al. critically discussed the major challenges, which may contribute to future studies on elucidating the mechanisms and developing novel therapies for FSGS. It has been shown that miRNAs regulate various cellular processes and immune responses (8). Nejad et al. provided new insights into the roles of miRNAs in multiple sclerosis, a potentially disabling autoimmune disorder with inflammation in central nervous system. The review discusses the involvement of miRNAs in MS pathogenesis and the underlying mechanisms, highlighting the therapeutic potential of targeting miRNAs for treating the disease.

This Research Topic establishes a platform for future studies on organ-specific immunity in autoimmune diseases. The topic provides novel findings on organ-specific immune dysregulations, new histopathological and serological examination methods, and potential effective combinational therapies. These studies will improve the current understanding of organ-specific autoimmune responses and facilitate future development of novel targeted immunotherapies.
