# Correction: Editorial: Advances in organ-specific autoimmune response: from basics to clinics

**DOI:** 10.3389/fimmu.2026.1926718

**Published:** 2026-07-15

**Authors:** Fan Xiao, Xuming Tang, Kongyang Ma, Xiaoyan Dai

**Affiliations:** 1 Department of Pathology, Li Ka Shing Faculty of Medicine, The University of Hong Kong, Hong Kong, Hong Kong SAR, China; 2 National Heart, Lung, and Blood Institute, National Institutes of Health, Bethesda, MD, United States; 3 Centre for Infection and Immunity Studies, School of Medicine, The Sun Yat-sen University, Guangdong, Shenzhen, China; 4 Clinical Research Institute, the Second Affiliated Hospital, Hengyang Medical School, University of South China, Hengyang, China

**Keywords:** autoimmune disease, organ-specific responses, pathogenesis, diagnosis, therapy

The **Author contributions** statement was erroneously given as: “FX: Writing – original draft. XT: Writing – review & editing, Funding acquisition. KM: Writing – review & editing, Funding acquisition. XD: Writing – review & editing, Funding acquisition”. The correct **Author contributions** statement is: “FX: Writing – original draft. XT: Writing – review & editing. KM: Writing – review & editing, Funding acquisition. XD: Writing – review & editing, Funding acquisition”.

The original version of this article has been updated.

